# Arf GTPases Define BST-2-Independent Pathways for HIV-1 Assembly and Release

**DOI:** 10.3390/v18010011

**Published:** 2025-12-20

**Authors:** Adam Smith, Dominique Dotson, Jessica Sutton, Hua Xie, Xinhong Dong

**Affiliations:** 1 Department of Microbiology, Immunology, and Physiology, School of Medicine, Meharry Medical College, Nashville, TN 37208, USA; 2 Center for AIDS Health Disparities Research, School of Medicine, Meharry Medical College, Nashville, TN 37208, USA; 3 School of Dentistry, Meharry Medical College, Nashville, TN 37208, USA

**Keywords:** HIV-1 Gag, ADP-ribosylation factor, Arf1, Arf6, virus assembly, membrane trafficking, BST-2, virion release

## Abstract

ADP-ribosylation factor (Arf) proteins are small GTPases that regulate intracellular membrane trafficking and actin remodeling through tightly controlled cycles of GTP binding and hydrolysis. Arf1, a central coordinator of Golgi and endosomal transport, and Arf6, which regulates plasma membranes and endosomal dynamics, have both been implicated in late stages of the HIV-1 life cycle. However, the mechanisms by which these GTPases support viral assembly and release remain incompletely defined. Here, we provide direct evidence that both Arf1 and Arf6 are required for efficient trafficking of the HIV-1 Gag polyprotein, assembly, and virion production. Perturbation of Arf1 function using either GTP-locked (Q71L) or GDP-locked (T31N) mutants significantly reduced virus release, impaired Gag association with membrane compartments, and prevented its accumulation at the plasma membrane. Manipulation of Arf1 cycling through the GTPase-activating protein AGAP1 further demonstrated that dynamic transitions between GTP- and GDP-bound states are essential for productive Gag trafficking. Similarly, expression of a constitutively active Arf6 mutant (Q67L) misrouted Gag to intracellular membranes and markedly suppressed virion release. Importantly, disruption of Arf1 or Arf6 activity did not affect the expression, surface levels, or intracellular distribution of the host restriction factor BST-2. Together, these findings identify Arf1- and Arf6-mediated trafficking pathways as critical host determinants of HIV-1 assembly and release and establish that their functions operate independently of BST-2 antagonism.

## 1. Introduction

The Gag protein is the major structural component of HIV-1 and other retroviruses, orchestrating virion assembly, release, and maturation [1,2,3]. Remarkably, Gag alone is sufficient to drive the formation and release of virus-like particles (VLPs) from the cell surface in the absence of other viral components. HIV-1 Gag is synthesized as a polyprotein precursor comprising four major structural domains: the N-terminal matrix (MA), capsid (CA), nucleocapsid (NC), and C-terminal p6 domains [1,4]. Each domain plays distinct roles during late stages of the viral life cycle [5,6]. The MA domain, through a N-terminal myristylation signal and a highly basic region (HBR), directs membrane targeting and binding [7,8]. The CA domain governs Gag–Gag interactions required for multimerization [9,10], while the NC domain binds and packages the viral RNA genome and also contributes to Gag multimerization during assembly [11]. The p6 domain recruits the host ESCRT (endosomal sorting complexes required for transport) machinery through interaction with Tsg101 and ALIX, thereby enabling membrane fission and virion release [12,13,14].

While the intrinsic biochemical properties of Gag are sufficient for particle formation in heterologous expression systems, increasing evidence highlights the importance of host cell trafficking pathways and cytoskeletal networks in regulating Gag localization and virion egress [15,16,17,18]. Small GTPases of the ADP-ribosylation factor (Arf) family are key regulators of membrane traffic and vesicle biogenesis, particularly in the context of Golgi and endosomal transport [19,20,21]. Arf proteins cycle between GDP-bound (inactive) and GTP-bound (active) states to coordinate cargo sorting, vesicle formation, and organelle identity [21,22]. Among these, Arf1 Arf6 have emerged as regulators of retroviral egress, although the precise mechanisms remain incompletely defined [23,24]. Arf1 predominantly localized to the Golgi and regulates vesicle trafficking via adaptor proteins such as the GGAs (Golgi-localized, γ-ear-containing, Arf-binding proteins), while Arf6 functions at the plasma membrane and endosomal compartments, controlling actin dynamics, endocytosis, and recycling [25,26]. Both Arf1 and Arf6 have been implicated in the release of retroviruses such as HIV-1, murine leukemia virus (MLV), and equine infectious anemia virus (EIAV), raising the possibly that Arf-mediated trafficking influences viral assembly or access to key egress factors [23].

One such egress factor is BST-2 (bone marrow stromal antigen 2; also known as tetherin or CD317), an interferon-inducible type II transmembrane protein that potently inhibits the release of HIV-1 and other enveloped viruses. BST-2 functions by physically tethering nascent virions to the plasma membrane or to each other, preventing their dissemination from infected cells [27,28,29]. Structurally, BST-2 consists of an N-terminal cytoplasmic tail, a single-pass transmembrane domain, a coiled-coil extracellular domain, and a C-terminal glycosylphosphatidylinositol (GPI) anchor [30]. This unique topology allows it to insert one membrane anchor into the plasma membrane and the other into budding virions, thereby forming a physical bridge that retains virions at the cell surface [27,31,32]. In response, HIV-1 encodes the accessory protein Vpu, which antagonizes BST-2 by promoting its degradation through both proteasomal and lysosomal pathways, sequestering it in intracellular compartments, and displacing it from viral budding sites [33,34,35]. The Vpu-BST-2 axis represents a key example of host–pathogen antagonism and serves as a model for understanding how viruses counteract intrinsic immunity.

While both Arf proteins and BST-2 have been independently linked to HIV-1 egress, it remains unclear whether Arf-mediated trafficking regulates the localization, stability, or antiviral function of BST-2. Moreover, whether the impact of Arf proteins on HIV-1 release is dependent on BST-2 antagonism or reflects parallel trafficking mechanisms required for Gag transport remains to be determined.

In this study, we investigated the role of Arf1 and Arf6 in modulating HIV-1 particle production, Gag subcellular localization, and BST-2 trafficking. Using biochemical, virological, and imaging approaches, we demonstrate that both Arf1 and Arf6 are required for efficient trafficking of HIV-1 Gag to the plasma membrane and for productive virus release. Disruption of either GTPase alters Gag trafficking, leading to intracellular accumulation and substantial reduction in VLP production. Importantly, these effects are independent of BST-2 expression or localization, suggesting that Arf1 and Arf6 promote HIV-1 assembly and egress through BST-2-independent pathways. These findings identify Arf GTPases as critical host factors co-opted by HIV-1 to support late stages of the viral life cycle, likely by directing Gag trafficking through distinct membrane compartments en route to the plasma membrane.

## 2. Materials and Methods

### 2.1. Cell Cultures and Transfections

HeLa (ATCC CCL2) and HEK 293T (ATCC CRL-3216) cells were obtained from the American Type Culture Collection (ATCC) and maintained in Dulbecco’s Modified Eagle’s Medium (DMEM; high glucose; Thermo Fisher Scientific, Waltham, MA, USA) supplemented with 2 mM L-glutamine, 10% fetal bovine serum (FBS; Thermo Fisher Scientific), 100 U/mL penicillin, and 100 μg/mL streptomycin at 37 °C in a humidified incubator with 5% CO_2_. HeLa cells were transfected using either Lipofectamine 2000 (Thermo Fisher Scientific) or TransIT-HeLaMONSTER (Mirus Bio, Madison, WI, USA) according to the manufacturers’ instructions. HEK 293T cells were transfected using either polyethyleneimine (PEI, Sigma-Aldrich, St. Louis, MO, USA), calcium phosphate precipitation, or X-tremeGENE HP DNA transfection reagent (Sigma-Aldrich).

### 2.2. Plasmids

An expression plasmid encoding HA-tagged full-length Arf1 was kindly provided by Juan Bonifacino. The following plasmids were obtained from Thomas Roberts via Addgene: pcDNA3-HA-Arf1/Q71L (plasmid #10800), encoding HA-tagged Arf1 mutation with Q71L substitution; pcDNA3-HA-Arf1/T31N (plasmid #10801), encoding HA-tagged Arf1 mutation with T31N substitution; pcDNA3-HA-Arf6 (plasmid # 10802), encoding HA-tagged full-length Arf6; and pcDNA3-HA-Arf6/Q67L (plasmid # 10803), encoding HA-tagged Arf6 mutation with Q67L substitution [36]. The HIV-1 Gag-Pol expression plasmid pGPCINS was provided by Xiao-Fang Yu [37], and the Gag-RFP construct was kindly provided by Akira Ono [38]. FLAG-tagged AGAP1 (WT and R599K mutant) constructs were obtained from Paul Randazzo [39].

### 2.3. Flow Cytometry

HeLa cells were co-transfected with GFP and either HA-tagged Arf1, Arf1/Q71L, Arf1/T31N, Arf6, or Arf6/Q67L expression constructs. At 48 h post-transfection, cells were stained with APC-conjugated anti-human BST-2 antibodies (BioLegend, San Diego, CA, USA, Cat. No. 348410) and analyzed on a Luminex Amnis CellStream flow cytometer. Flow cytometric data were processed and quantified using FlowJo v11 software (BD Biosciences).

### 2.4. Immunofluorescence Microscopy

Confocal immunofluorescence microscopy was performed as described previously [40]. For single-staining experiments, HA- or FLAG-tagged proteins were detected using mouse anti-HA (Abcam, Waltham, MA, USA, Cat. No. 1424) or anti-FLAG (Sigma-Aldrich, Cat. No. F3165) primary antibodies, followed by Alexa Fluor 488-conjugated goat anti-mouse secondary antibodies (Thermo Fisher Scientific, Cat. No. A-11001). For double-staining experiments, Alexa Fluor 546-conjugated goat anti-mouse secondary antibodies (Thermo Fisher Scientific, Cat. No. A-11003) were used to visualize HA- or FLAG-tagged proteins. Endogenous BST-2 was detected using rabbit anti-BST-2 antibodies (Abcam, Cat. No. ab243230) and Alexa Fluor 488-conjugated goat anti-rabbit secondary antibodies (Thermo Fisher Scientific, Cat. No. A-11008). HIV-1 Gag was detected using rabbit anti-p17 antibodies (BEI Resources, Manassas, VA, USA, Cat. No. ARP-4811) and Alexa Fluor 488-conjugated goat anti-rabbit secondary antibodies. Images were acquired using a Nikon A1R confocal microscope and analyzed with NIS-Elements AR 5.20.01 64-bit software (Nikon Instruments Inc., Tokyo, Japan).

Pearson’s correlation coefficients were calculated to quantify colocalization between fluorescence signals, based on analyses of 20–30 cells per condition from at least three independent experiments. Pearson correlation coefficients (*r*) were calculated using the standard formula:

$$r=\frac{\sum_{i=1}^{n}\left(x_{i}-\bar{x}\right)\left(y_{i}-\bar{y}\right)}{\sqrt{\sum_{i=1}^{n}{\left(x_{i}-\bar{x}\right)}^{2}}\sqrt{\sum_{i=1}^{n}{\left(y_{i}-\bar{y}\right)}^{2}}}$$

where $x_{i}$ and $y_{i}$ represent the fluorescence intensities of individual pixels in channels X and Y, and $\bar{x}$ and $\bar{y}$ denote their respective mean intensities.

### 2.5. Virion/VLP Production and Purification

HEK 293T cells were co-transfected with HIV-1 NL4-3 or HIV-1 Gag-Pol and either empty vector (pcDNA3.1), HA-Arf1, HA-Arf1/Q71L, HA-Arf1/T31N, FLAG-AGAP1, or HA-Arf6/Q67L. At 48 h post-transfection, culture supernatants were collected, clarified by centrifugation at 500× *g* for 10 min, passed through a 0.45 µm filter, and pelleted through a 20% sucrose cushion at 28,000× *g* for 2 h at 4 °C. The resulting virion or VLP pellets were resuspended and analyzed by Western blot.

### 2.6. Membrane Flotation Assay

A modified membrane flotation assay was used to assess HIV-1 Gag membrane association [41]. HEK 293T cells were co-transfected with HIV-1 Gag and either pcDNA3.1, HA-Arf1, HA-Arf1/Q71L, or HA-Arf1/T31N. At 48 h post-transfection, cells were harvested using trypsin-EDTA, resuspended in hypotonic buffer (10 mM Tris-HCl, pH 8.0, supplemented with protease inhibitors), and incubated on ice for 15 min. Cells were lysed by Dounce homogenization, and lysates were adjusted to 0.1 M NaCl. After clearing by centrifugation at 1000× *g* for 10 min at 4 °C, the post-nuclear supernatant was adjusted to 50% (*v*/*v*) iodixanol from a 60% stock (Axis-Shield, Oslo, Norway) and overlaid with 40%, 30%, and 10% iodixanol solutions prepared in buffer (0.85% NaCl, 10 mM Tricine-NaOH, pH7.4). Gradients were centrifuged at 35,000 rpm for 16 h at 4 °C in an SW41Ti rotor (Beckman Coulter, Brea, CA, USA). Fractions were collected from the top and analyzed by Western blot using anti-p24 (BEI Resources, Manassas, VA, USA, Cat. No. ARP-3537) and anti-HA (Abcam, Cat. No. ab18181) antibodies.

### 2.7. Statistical Analysis

Quantitative data are presented as mean ± standard deviation (SD) from at least three independent experiments unless otherwise noted. Statistical significance was determined using unpaired two-tailed Student’s *t*-tests in GraphPad Prism v10 (GraphPad Software, San Diego, CA, USA). Statistical significance of correlations was assessed using two-tailed unpaired *t*-tests, with *p* < 0.05 considered significant.

## 3. Results

### 3.1. Arf1 Regulates HIV-1 Particle Release and Gag Subcellular Localization

To define the role of the small GTPase Arf1 in HIV-1 particle production, HEK 293T cells were co-transfected with plasmids encoding HIV-1 Gag-Pol and either an empty vector (pcDNA3.1), wild-type (WT) HA-tagged Arf1, a constitutively active GTP-locked mutant (HA-Arf1/Q71L), or a dominant-negative GDP-locked mutant (HA-Arf1/T31N). At 48 h post-transfection, cell lysates and pelleted virus-like particles (VLPs) from clarified supernatants were harvested. Viral proteins were detected by immunoblotting using HIV IG (polyclonal anti-HIV immune globulin; BEI Resources, Cat. No. ARP-3957) to probe for Gag.

**Figure 1:**
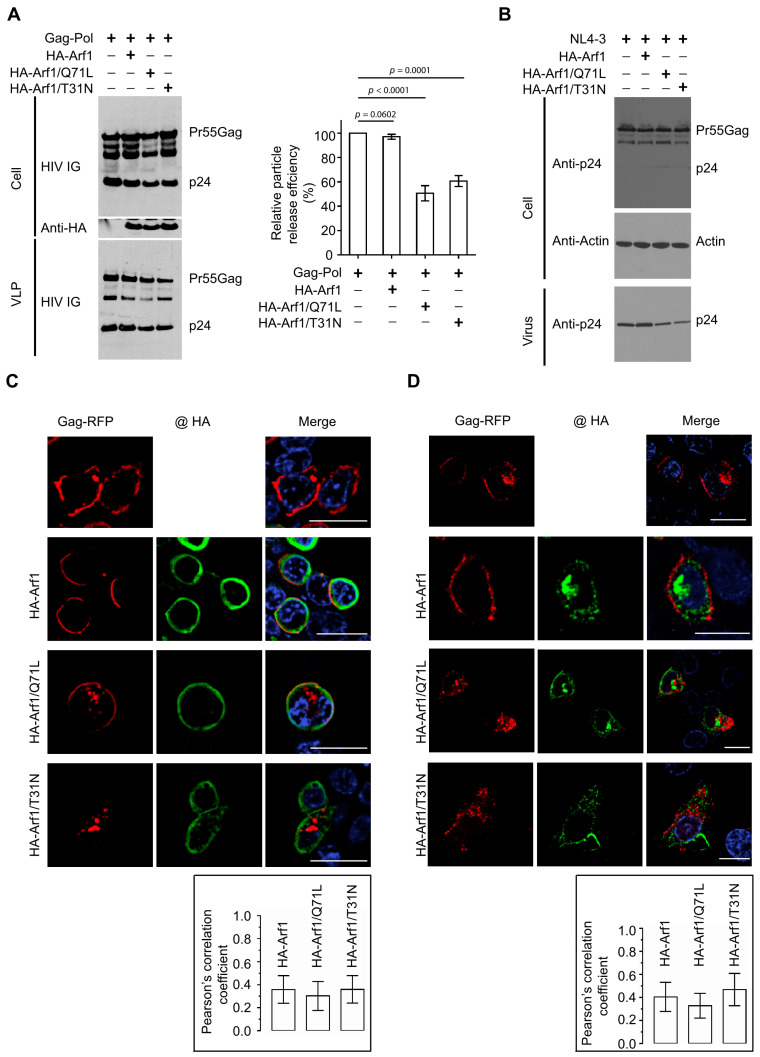
Arf1 regulates HIV-1 particle production and subcellular localization of Gag. (**A**) HEK 293T cells were co-transfected with HIV-1 Gag-Pol and either empty vector (pcDNA3.1; lane 1), WT HA-Arf1 (lane 2), HA-Arf1/Q71L (lane 3), or HA-Arf1/T31N (lane 4) at a 1:1 plasmid ratio. Cell lysates were analyzed by immunoblotting using HIV IG (polyclonal anti-HIV immune globulin) and anti-HA antibodies, and VLPs pelleted from culture supernatants were probed with HIV IG. The right panel shows quantification of particle release efficiency, calculated as the ratio of Gag in the supernatant to total Gag (supernatant plus cell-associated) and normalized to the pcDNA3.1 control. Data represent the mean ± SD from six independent experiments (n = 6). *p* values were determined using a two-tailed unpaired *t*-test. (**B**) HEK 293T cells were co-transfected with HIV-1 NL4-3 and either empty vector (pcDNA3.1; lane 1), WT HA-Arf1 (lane 2), HA-Arf1/Q71L (lane 3), or HA-Arf1/T31N (lane 4) at a 1:1 plasmid ratio. Cell lysates were analyzed by immunoblotting with anti-p24 and anti-HA antibodies, and virions pelleted from supernatants were probed using anti-p24 antibodies. Blots shown are representative of three independent experiments. (**C**,**D**) Confocal microscopy of HEK 293T (**C**) or HeLa (**D**) cells expressing Gag-RFP alone (**top row**), Gag-RFP with HA-Arf1 (**second row**), Gag-RFP with HA-Arf1/Q71L (**third row**), or Gag-RFP with HA-Arf1/T31N (**bottom row**). Gag-RFP is shown in red (**left panels**), HA-tagged proteins in green (**middle panels**), and merged images in yellow (**right panels**). Scale bars, 20 μm. Pearson’s correlation coefficient (*R*) were used to quantify colocalization. Graphs show the mean ± SD from 20–30 cells per condition based on ≥3 independent experiments.

Overexpression of WT Arf1 did not appreciably alter Gag expression, processing, or VLP release relative to the vector control. In contrast, expression of either Arf1/Q71L or Arf1/T31N moderately but reproducibly reduced VLP release (Figure 1A, left), indicating that dynamic cycling between GTP- and GDP-bound states is required for efficient HIV-1 particle production. VLP release efficiency was calculated as the ratio of VLP-associated Gag to total Gag (cell-associated plus VLP-associated) and normalized to the vector control (Gag-Pol + pcDNA3.1, lane 1). Consistent with the qualitative observations, expression of either Arf1/Q71L or Arf1/T31N modestly decreased release efficiency, whereas WT Arf1 had no detectable effect (Figure 1A, right). These data suggest that both impaired GTP hydrolysis (Q71L) and defective GTP loading (T31N) compromise Arf1 function during HIV-1 assembly and/or release.

To confirm these findings, we repeat the experiments using the full-length HIV-1 molecular clone NL4-3 in place of the Gag-Pol plasmid. Western blot analysis of cell and virion lysates using anti-p24 antibodies demonstrated that expression of either Arf1/Q71L or Arf1/T3N similarly reduced virus release, further supporting the conclusion that perturbations in Arf1 activity impair HIV-1 particle production (Figure 1B).

To examine how Arf1 activity affects Gag trafficking, confocal microscopy was performed in HEK 293T cells co-expressing Gag-RFP and HA-tagged Arf1 constructs. In cells expressing Gag-RFP alone, the protein localized predominantly to the plasma membrane, consistent with its role in viral assembly. Co-expression of WT HA-Arf1 did not alter this pattern; Gag-RFP remained largely membrane-associated, and minimal colocalization was observed with Arf1, which localized mainly to sub-membranous regions. In contrast, expression of either HA-Arf1/Q71L or HA-Arf1/T31N disrupted Gag-RFP trafficking, reducing its accumulation at the plasma membrane and increasing its presence in perinuclear compartments (Figure 1C). Both Arf1 mutants displayed sub-membranous localization patterns similar to WT Arf1. Quantitative colocalization analysis using Pearson’s correlation coefficients revealed limited overlap between Gag-RFP and HA-Arf1 under all tested conditions (WT, 0.36 ± 0.12; Q71L, 0.30 ± 0.13; T31N, 0.36 ± 0.12).

To validate these observations in a cell type with more defined membrane architecture, parallel analyses were performed in HeLa cells. In control cells, Gag-RFP localized to both the plasma membrane and internal compartments. Expression of either Arf1/Q71L or Arf1/T31N markedly reduced plasma membrane-associated Gag-RFP and enhanced its accumulation in perinuclear regions (Figure 1D), consistent with findings in HEK 293T cells. Pearson’s correlation coefficients for HeLa cells were similarly low to moderate (WT, 0.40 ± 0.12; Q71L, 0.33 ± 0.11; T31N, 0.46 ± 0.14). To confirm these results independently of the RFP tag, untagged Gag was examined by anti-p17 (MA) immunostaining. Gag alone localized predominantly to the plasma membrane at 24 h post-transfection, whereas co-expression of either HA-Arf1/Q71L or HA-Arf1/T31N caused redistribution to internal membranes with reduced plasma membrane localization (Figure S1).

Together, these findings demonstrate that perturbation of Arf1 GTPase cycling impairs Gag trafficking and reduces HIV-1 particle release, underscoring a functional requirement for dynamic Arf1 activity during the late stages of viral assembly and budding.

### 3.2. Arf1 Regulates the Membrane Association of HIV-1 Gag

To determine whether Arf1 influences the membrane association of HIV-1 Gag, membrane flotation assays were performed using discontinuous iodixanol gradients. HEK 293T cells were co-transfected with plasmids encoding HIV-1 Gag and either empty vector (pcDNA3.1), WT HA-Arf1, HA-Arf1/Q71L, or HA-Arf1/T31N. At 48 h post-transfection, cells were harvested, lysed in hypotonic buffer, and post-nuclear supernatants were subjected to ultracentrifugation on 10–50% iodixanol step gradients. Twelve fractions were collected from top to bottom, corresponding to low-density (membrane-associated) and high-density (cytosolic or membrane-free) components, and analyzed by immunoblotting using anti-p24 to detect Gag and anti-HA to detect Arf1 variants.

**Figure 2:**
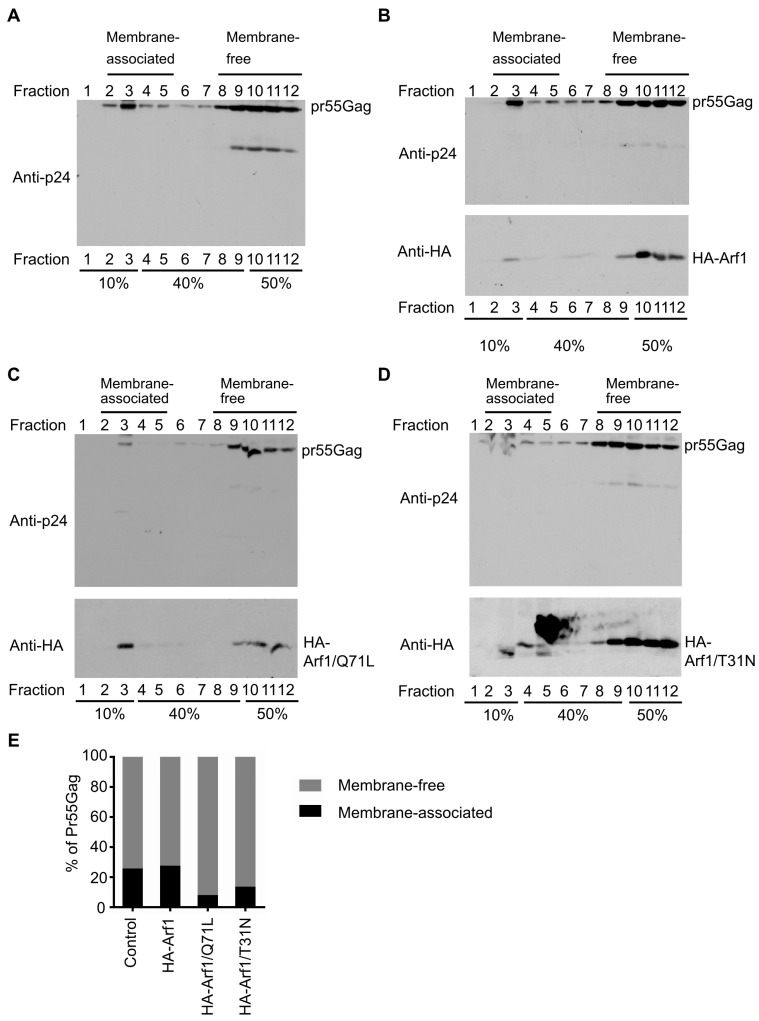
Arf1 regulates the membrane association of HIV-1 Gag. (**A**–**D**) HEK 293T cells were co-transfected with HIV-1 Gag and either (**A**) empty vector (pcDNA3.1), (**B**) WT HA-Arf1, (**C**) HA-Arf1/Q71L, or (**D**) HA-Arf1/T31N. At 48 h post-transfection, cells were lysed and subjected to membrane flotation centrifugation. Twelve equal-volume fractions were collected from the top of the gradient and analyzed by immunoblotting with anti-p24 and anti-HA antibodies. Factions 2–5 correspond to membrane-associated proteins, whereas fractions 8–12 represent cytosolic (membrane-free) proteins. (**E**) Quantification of membrane-associated versus membrane-free Gag under each condition. Bar graphs represent the percentage of total Gag detected in membrane-associated (fractions 2–5) and membrane-free (fractions 8–12) fractions. Data are representative of three independent experiments.

In cells expressing Gag alone, the protein was distributed across both membrane-associated fractions (fractions 2–5) and denser, membrane-free fractions (fractions 8–12) (Figure 2A). Co-expression with WT HA-Arf1 did not markedly alter this pattern (Figure 2B). In contrast, expression of either HA-Arf1/Q71L or HA-Arf1/T31N caused a pronounced shift of Gag from membrane-associated fractions to denser fractions (Figure 2C,D), indicating impaired membrane targeting. Immunoblotting of total lysates confirmed comparable expression of Gag and Arf1 constructs across all conditions (Figure 1A), demonstrating that the altered distribution was not attributable to differences in protein abundance.

Quantitative analysis showed that the proportion of Gag in membrane-associated fractions (fractions 2–5) was reduced by approximately 50% in cells expressing either Arf1 mutant compared with vector or WT Arf1 controls (Figure 2E). This reduction correlated with the decreased VLP release efficiency observed in parallel experiments (Figure 1A), suggesting that Arf1 promotes HIV-1 egress by facilitating Gag association with appropriate membrane compartments. The observation that both the GTP-locked (Q71L) and GDP-locked (T31N) mutants impaired Gag membrane association supports the model that dynamic cycling between GTP- and GDP-bound states is essential for Arf1 function during viral assembly.

### 3.3. AGAP1 Modulates HIV-1 Particle Release and Gag Trafficking

**Figure 3:**
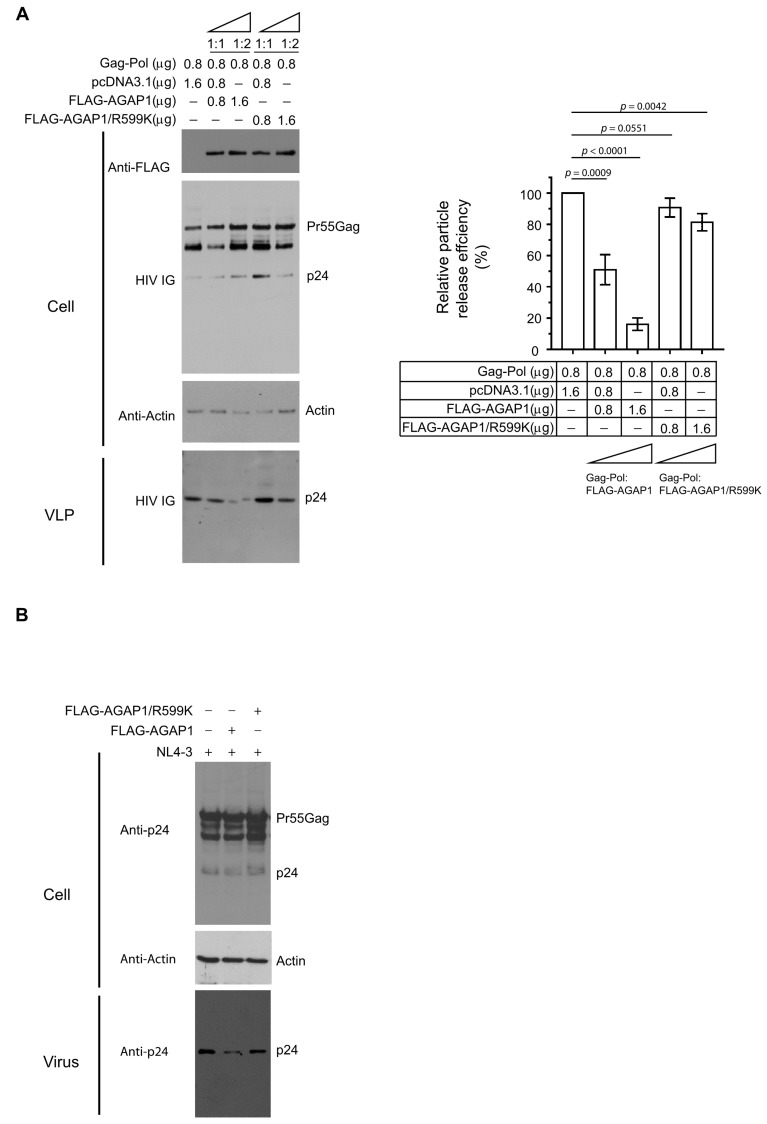

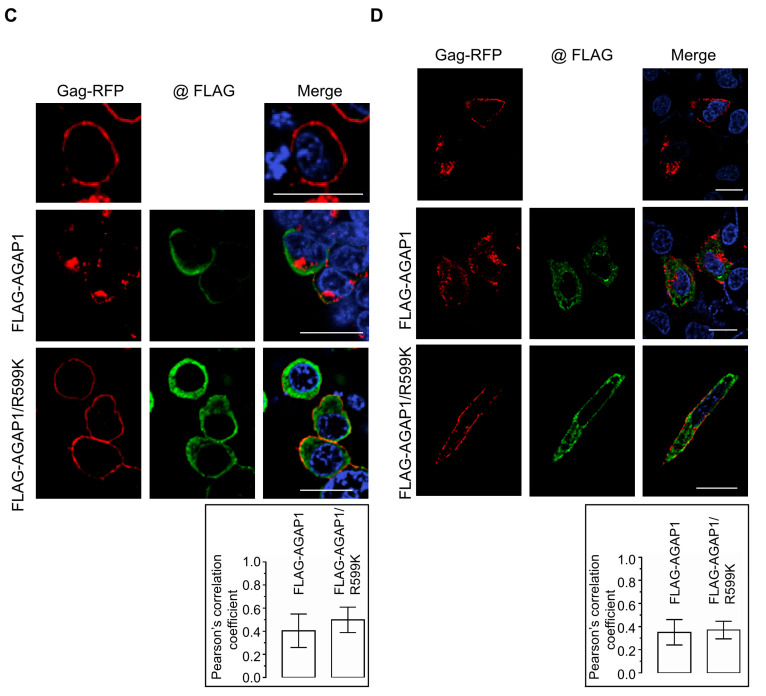
AGAP1 modulates HIV-1 particle release. (**A**) HEK 293T cells were co-transfected with HIV-1 Gag-Pol and either WT FLAG-AGAP1 (lanes 2–3), or FLAG-AGAP1/R599K (lanes 4–5) at increasing plasmid ratios (from 1:0 to 1:2 relative to Gag-Pol). Empty vector (pcDNA3.1) was added to normalize total DNA across samples. Cell lysates were analyzed by immunoblotting with anti-FLAG, HIV IG, and anti-actin antibodies, and pelleted VLPs were probed with HIV IG. The **right panel** shows quantification of particle release efficiency, calculated as the ratio of Gag in the VLP fraction to total Gag (VLP plus cell-associated) and normalized to the control. Data represent the mean ± SD from three independent experiments (n = 3). *p* values were calculated using a two-tailed unpaired *t*-test. (**B**) HEK 293T cells were co-transfected with HIV-1 NL4-3 and either WT FLAG-AGAP1 (lane 2) or FLAG-AGAP1/R599K (lane 3) at a 1:1 plasmid ratio. Empty vector (pcDNA3.1) was added to equalize total DNA input. Cell lysates were analyzed by immunoblotting using anti-p24 and anti-actin antibodies, and pelleted virions were probed with anti-p24 antibodies. Blots shown are representative of three independent experiments. (**C**,**D**) Confocal microscopy of HEK 293T (**C**) and HeLa (**D**) cells expressing Gag-RFP alone (**top row**), Gag-RFP with FLAG-AGAP1 (**second row**), or Gag-RFP with FLAG-AGAP1/R599K (**bottom row**). Gag-RFP is shown in red (**left panels**); FLAG-tagged proteins are shown in green (**middle panels**) by anti-FLAG immunostaining and fluorescent secondary antibodies; merged images appear in yellow (**right panels**). Scale bars, 20 μm. Colocalization was quantified using Pearson’s correlation coefficient (*R*). Graphs represent the mean ± SD from 20–30 cells per condition based on ≥3 independent experiments.

Because Arf1 activity is controlled by GTPase-activating proteins (GAPs), we next examined the role of AGAP1, an Arf1-specific GAP, in HIV-1 particle production [39,42]. HEK 293T cells were co-transfected with plasmids encoding HIV-1 Gag-Pol and either WT FLAG-tagged AGAP1 or a GAP-deficient mutant (FLAG-AGAP1/R599K) at plasmid ratios of 1:1 or 1:2 relative to Gag-Pol, with total DNA normalized using empty vector (pcDNA3.1). Immunoblot analysis of cell lysates and pelleted VLPs revealed that WT AGAP1 expression reduced VLP release in a dose-dependent manner (Figure 3A, left), consistent with the notion that excessive Arf1 inactivation interferes with HIV-1 assembly or budding. In contrast, expression of the catalytically inactive AGAP1/R599K mutant had minimal effect on VLP production (Figure 3A, lanes 4–5 vs. lane 1). Quantification of Gag in cells and supernatants confirmed a significant reduction in VLP release efficiency in cells expressing WT AGAP1 but not AGAP1/R599K (Figure 3A, right). Similarly, WT AGAP1, but not AGAP1/R599K, inhibited the release of HIV-1 NL4-3 (Figure 3B). These findings support a model in which AGAP1 regulates HIV-1 egress by promoting proper Arf1 GTPase cycling, and that disruption of this activity impairs particle release.

To determine whether AGAP1 influences intracellular Gag trafficking, Gag-RFP localization was examined in cells co-expressing AGAP1 constructs. In HEK 293T cells, expression of WT FLAG-AGAP1 caused a pronounced redistribution of Gag-RFP from the plasma membrane to intracellular compartments, frequently enriched in perinuclear regions. By contrast, co-expression of FLAG-AGAP1/R599K did not substantially alter Gag-RFP localization, which remained predominantly at the plasma membrane (Figure 3C). Quantitative colocalization analysis revealed no significant difference between WT and mutant AGAP1 (Pearson’s *R*: 0.40 ± 0.14 for WT, 0.49 ± 0.10 for R599K). Similar results were obtained in HeLa cells: WT AGAP1 disrupted Gag-RFP localization to the plasma membrane, whereas AGAP1/R599K had minimal effect (Figure 3D). Colocalization between Gag-RFP and AGAP1 constructs remained low and comparable (Pearson’s *R*: WT, 0.35 ± 0.11; R599K, 0.37 ± 0.07).

Parallel experiments using untagged Gag detected by anti-p17 (MA) immunostaining yielded consistent results: expression of WT AGAP1, but not the R599K mutant, reduced plasma membrane-associated Gag and promoted its accumulation at internal compartments (Figure S2).

Collectively, these findings demonstrate that AGAP1 modulates Gag trafficking through its catalytic GAP activity rather than through direct or stable interaction with Gag. The inability of the GAP-deficient mutant to alter Gag localization underscores the requirement for spatially and temporally regulated Arf1 inactivation during late stages of the HIV-1 replication cycle. Together, these results establish AGAP1 as a negative regulator of HIV-1 particle release through an Arf1 GAP activity-dependent mechanism.

### 3.4. Arf6 Modulates HIV-1 Particle Release and Gag Subcellular Localization

**Figure 4:**
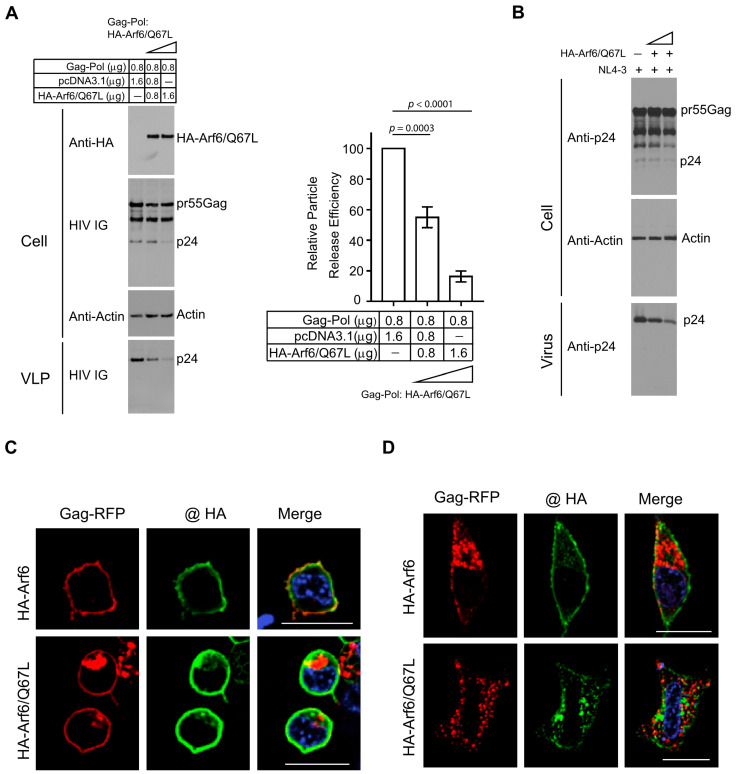
Arf6 modulates HIV-1 particle release. (**A**) HEK 293T cells were co-transfected with HIV-1 Gag-Pol alone (lane 1) or with HA-Arf6/Q67L at increasing DNA ratios (lanes 2 and 3; 1:1 and 1:2, Gag-Pol:Arf6/Q67L). Total DNA input was equalized using empty vector (pcDNA3.1). Cell lysates were analyzed by immunoblotting with anti-HA, HIV IG and anti-actin antibodies, and pelleted VLPs were probed with HIV IG. The **right panel** shows quantification of particle release efficiency, normalized to the control (lane 1). Data represent the mean ± SD from three independent experiments (n = 3). *p* values are indicated. (**B**) HEK 293T cells were co-transfected with HIV-1 NL4-3 alone (lane 1) or with HA-Arf6/Q67L at increasing DNA ratios (lanes 2 and 3; 1:1 and 1:2, NL4-3: Arf6/Q67L). Total DNA input was equalized using empty vector (pcDNA3.1). Cell lysates were analyzed by immunoblotting with anti-p24 and anti-actin antibodies, and pelleted virions were probed with anti-p24 antibodies. Blots shown are representative of three independent experiments. (**C**,**D**) Confocal fluorescence microscopy of HEK 293T (**C**) and HeLa (**D**) cells co-expressing Gag-RFP (red) and either WT HA-Arf6 (**top row**) or HA-Arf6/Q67L (**bottom row**). HA-tagged proteins were detected by immunostaining with anti-HA antibodies and Alexa Fluor-conjugated secondary antibodies (green). Merged images show colocalization in yellow. Scale bars, 20 μm.

Arf6 is a small GTPase involved in endosomal membrane trafficking and actin cytoskeleton remodeling, and it has been implicated in the late stages of the HIV-1 replication cycle through regulation of Gag trafficking [24]. To further define the role of Arf6 in HIV-1 particle assembly and release, HEK 293T cells were co-transfected with HIV-1 Gag-Pol and a constitutively active, GTP-locked mutant of Arf6 (HA-Arf6/Q67L) at increasing DNA ratios (1:1 and 1:2 relative to Gag-Pol), with empty vector (pcDNA3.1) added to normalize total DNA input. Immunoblot analysis of cell lysates and pelleted VLPs revealed a dose-dependent reduction in extracellular VLP production upon HA-Arf6/Q67L expression. Notably, intracellular levels of Pr55Gag and its processing intermediates were unaffected, indicating that Arf6 activation impairs a post-translational step—likely involving Gag trafficking or particle release—rather than Gag expression or proteolytic processing (Figure 4A, left). Quantification of VLP release efficiency confirmed a significant reduction in particle release in the presence of activated Arf6 (Figure 4A, right). A similar inhibitory effect of HA-Arf6/Q67L on virus release was observed in HEK 293T cells transfected with HIV-1 NL4-3 (Figure 4B). Together, these results suggest that constitutive Arf6 activation interferes with a late stage in HIV-1 assembly or budding.

To evaluate the impact of Arf6 on Gag subcellular localization, confocal fluorescence microscopy was performed in HEK 293T co-expressing Gag-RFP and either WT HA-Arf6 or HA-Arf6/Q67L. At 24 h post-transfection, WT HA-Arf6 colocalized strongly with Gag-RFP at the plasma membrane without altering Gag distribution (Figure 4C). In contrast, expression of HA-Arf6/Q67L caused a pronounced redistribution of Gag-RFP to intracellular puncta, consistent with retention in endosomal or recycling compartments. Similar results were observed in HeLa cells, where WT HA-Arf6 co-expression supported Gag-RFP localization at both the plasma membrane and intracellular compartments, whereas HA-Arf6/Q67L expression led to predominant intracellular accumulation and reduced plasma membrane association (Figure 4D). Parallel experiments using untagged Gag confirmed that Arf6/Q67L expression impaired Gag trafficking to the plasma membrane (Figure S3).

Collectively, these findings demonstrate that constitutively active Arf6 perturbs Gag trafficking and membrane targeting, thereby disrupting efficient HIV-1 particle assembly and release.

### 3.5. Arf1-Mediated Trafficking Pathways Do Not Alter BST-2 Subcellular Localization or Surface Expression

The antiviral activity of BST-2 depends on its subcellular localization. Under steady-state conditions, BST-2 is distributed between the plasma membrane and intracellular compartments, including the trans-Golgi network (TGN) and recycling endosomes. Because Arf1 regulates vesicular transport between the Golgi and endosomal systems, we examined whether modulation of Arf1 activity affects the localization of endogenous BST-2.

**Figure 5:**
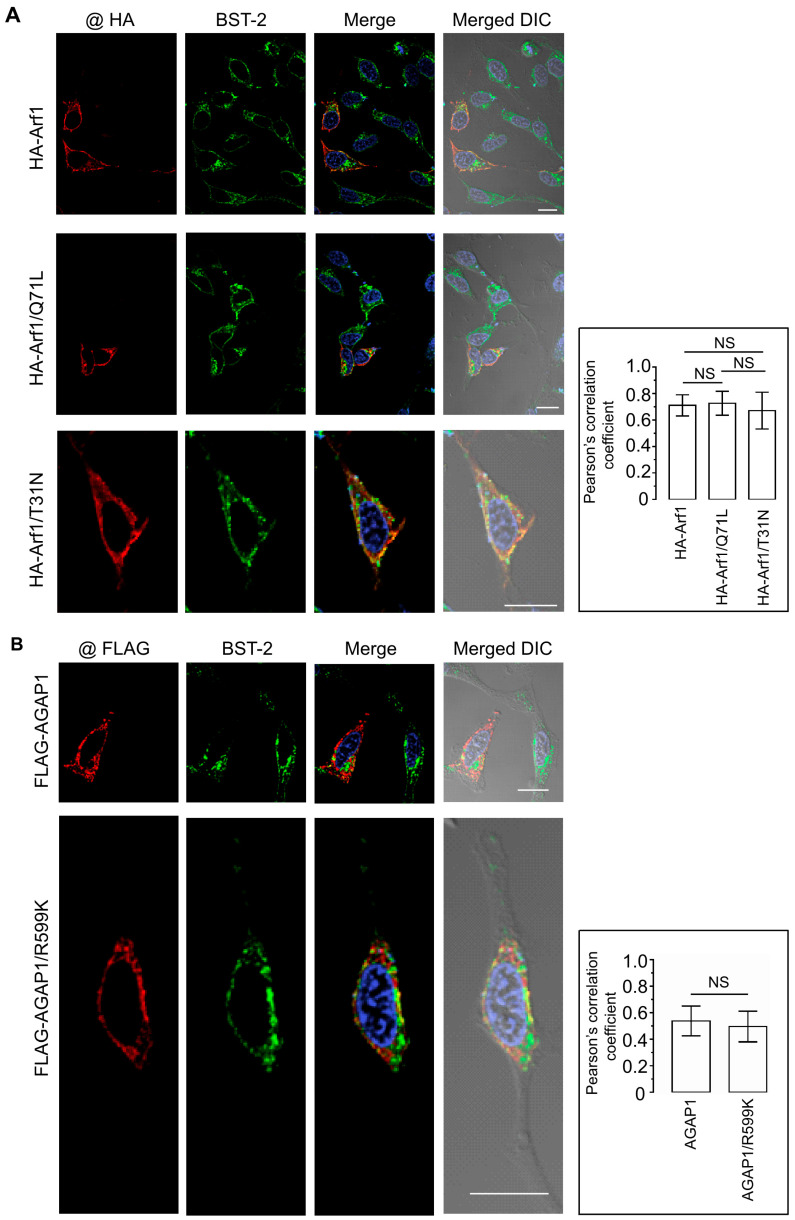
Disruption of Arf1 function does not alter BST-2 subcellular distribution. (**A**) HeLa cells were transfected with HA-Arf1 (**top row**), HA-Arf1/Q71L (**middle row**), or HA-Arf1/T31N (**bottom row**). At 30 h post-transfection, cells were fixed, permeabilized, and stained with anti-HA and anti-BST-2 antibodies. HA-tagged proteins are shown in red (**far-left panels**), endogenous BST-2 in green (**left panels**), and colocalized pixels in yellow (**right panels**). Differential interference contrast (DIC) images merged with confocal images are shown in the far-right panels. The graph shows Pearson’s correlation coefficients quantifying colocalization between HA-tagged proteins and endogenous BST-2. Data represent the mean ± SD from 20–25 cells based on at least three independent experiments. Scale bars, 20 μm. (**B**) HeLa cells were transfected with FLAG-AGAP1 (**top row**) or FLAG-AGAP1/R599K (**bottom row**) and immuno-stained with anti-FLAG and anti-BST-2 antibodies. FLAG-tagged proteins are shown in red (**far-left panels**), BST-2 in green (**middle panels**), and colocalization in yellow (**right panels**). DIC images merged with confocal images are shown in the far-right panels. Pearson’s correlation coefficients for colocalization between FLAG-tagged proteins and BST-2 are shown in the accompanying graph. Data represent the mean ± SD from 20–25 cells based on at least three independent experiments. Scale bars, 20 μm.

HeLa cells were transfected with HA-tagged constructs encoding WT Arf1, Arf1/Q71L, or Arf1/T31N. Cells were subsequently analyzed by immunofluorescence microscopy using antibodies against the HA epitope and endogenous BST-2. In all conditions, BST-2 exhibited its characteristic punctate distribution with enrichment at the plasma membrane and a perinuclear region consistent with the TGN. Each Arf1 construct showed substantial colocalization with BST-2-positive compartments (Figure 5A). Quantification of Pearson’s correlation coefficients from more than 30 cells per condition yielded values of 0.71 ± 0.08 for WT Arf1, 0.73 ± 0.09 for Arf1/Q71L, and 0.67 ± 0.14 for Arf1/T31N. No statistically significant differences were detected, indicating that Arf1 activity does not influence BST-2 subcellular distribution.

To assess whether AGAP1 affects BST-2 localization, HeLa cells were transfected with FLAG-tagged WT AGAP1 or a mutant (R599K) and stained for FLAG and endogenous BST-2. Both AGAP1 constructs exhibited a perinuclear localization and partial overlap with BST-2 (Figure 5B). Nevertheless, BST-2 maintained its distribution between the plasma membrane and TGN under both conditions. Pearson’s correlation coefficients were 0.54 ± 0.11 for WT AGAP1 and 0.50 ± 0.11 for the R599K mutant, with no significant difference between groups.

**Figure 6:**
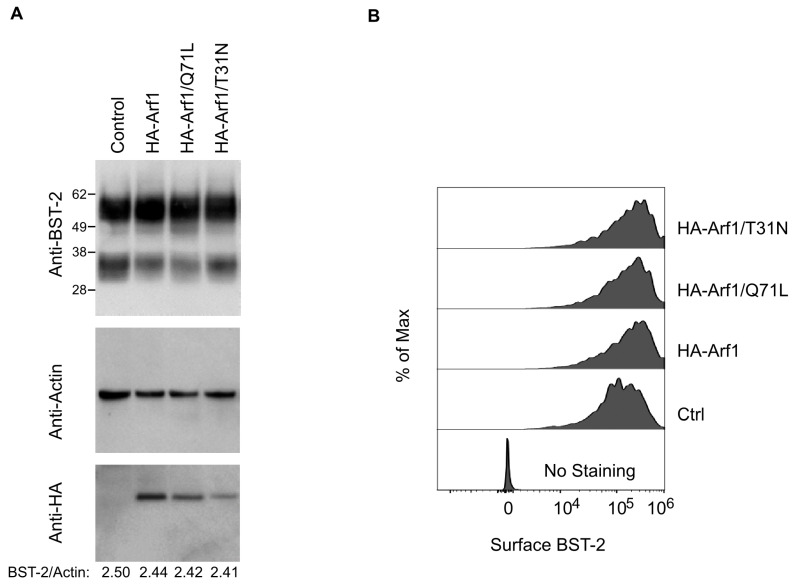
Impairment of Arf1 function does not change BST-2 expression. (**A**) HeLa cells were transfected with pcDNA3.1 (lane 1), HA-Arf1 (lane 2), HA-Arf1/Q71L (lane 3), or HA-Arf1/T31N (lane 4). Cell lysates were analyzed by immunoblotting with anti-HA, anti-BST-2, and anti-actin antibodies. Densitometric quantification of BST-2 levels normalized to actin is shown below the blots. Data are representative of three independent experiments. (**B**) HeLa cells were co-transfected with GFP and either pcDNA3.1, HA-Arf1, HA-Arf1/Q71L, or HA-Arf1/T31N. Surface expression of BST-2 on GFP-positive cells was assessed by flow cytometry.

To determine whether Arf1 modulates total or surface BST-2 expression, we performed immunoblotting of whole-cell lysates and flow cytometric analysis of surface BST-2 in transfected HeLa cells. Immunoblotting revealed no changes in total BST-2 levels upon expression of Arf1 WT, Q71L, or T31N, normalized to actin (Figure 6A). Flow cytometric analysis of GFP-positive cells co-transfected with Arf1 constructs confirmed that surface BST-2 expression remained comparable to empty vector controls (Figure 6B).

Together, these data demonstrate that neither Arf1 activity state nor AGAP1 expression significantly affects BST-2 subcellular localization, surface abundance, or total protein levels. Thus, the Arf1/AGAP1 regulatory axis does not appear to modulate BST-2 antiviral activity through alterations in its intracellular trafficking or membrane distribution.

### 3.6. Arf6 Does Not Modulate BST-2 Localization or Expression

**Figure 7:**
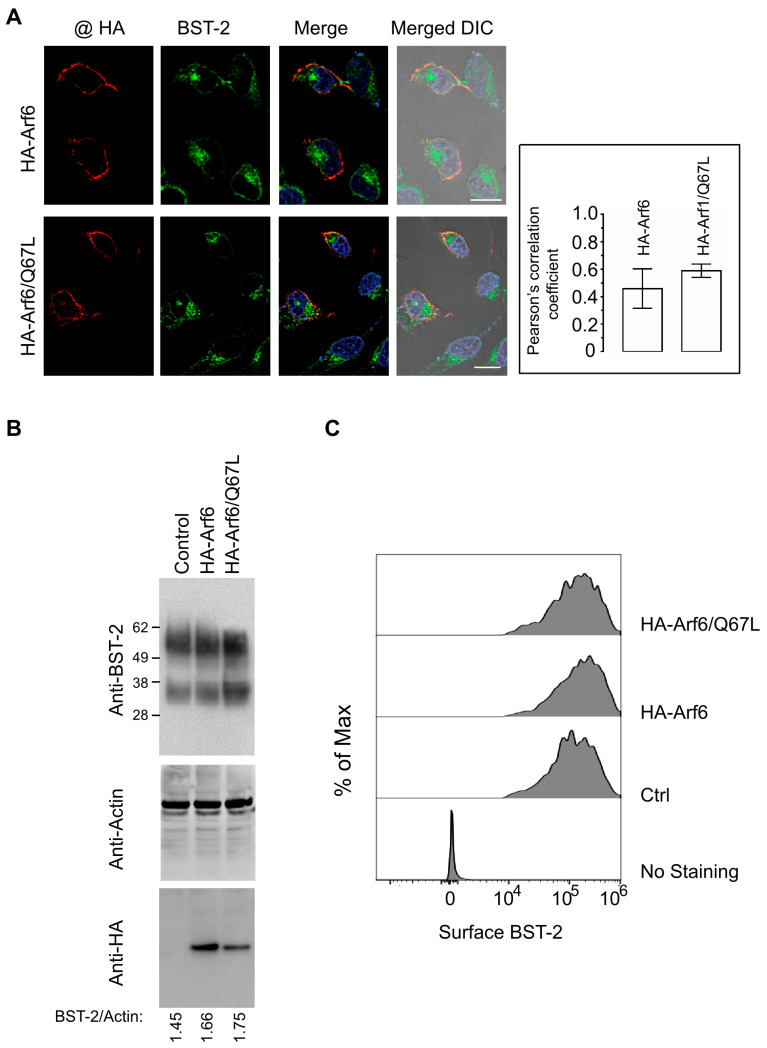
Arf6 function does not affect BST-2 expression or localization. (**A**) HeLa cells were transfected with HA-Arf6 (**top row**) or HA-Arf6/Q67L (**bottom row**), fixed, permeabilized, and stained with anti-HA and anti-BST-2 antibodies. Confocal images show HA-tagged proteins in red (**far-left panels**), BST-2 in green (**left panels**), and colocalized pixels in yellow (**right panels**). DIC images merged with fluorescence channels are shown in the far-right panels. The graph shows Pearson’s correlation coefficients quantifying colocalization between HA-tagged proteins and BST-2. Data represent the mean ± SD from 20–25 cells based on three independent experiments. (**B**) HeLa cells were transfected with pcDNA3.1 (lane 1), HA-Arf6 (lane 2), or HA-Arf6/Q67L (lane 3). Cell lysates were analyzed by immunostaining with anti-BST-2, anti-actin, and anti-HA antibodies. Densitometric ratios of BST-2 signal intensities normalized to corresponding actin levels are shown below the blots. Data are representative of three independent experiments. (**C**) HeLa cells were co-transfected with GFP and either pcDNA3.1, HA-Arf6, or HA-Arf6/Q67L. Surface expression of BST-2 on GFP-positive cells was assessed by flow cytometry.

To determine whether Arf6 affects BST-2 localization or expression, HeLa cells were transfected with HA-tagged WT Arf6 or Arf6/Q67L. At 30 h post-transfection, cells were analyzed by confocal immunofluorescence microscopy using antibodies against HA and endogenous BST-2. Both HA-Arf6 and HA-Arf6/Q67L displayed cytoplasmic localization with enrichment at the plasma membrane and perinuclear regions, partially overlapping with BST-2. However, neither construct caused a detectable redistribution of BST-2 from its characteristic localization at the TGN, recycling endosomes, and cell surface (Figure 7A). Quantitative colocalization analysis revealed moderate overlap between Arf6 and BST-2. WT HA-Arf6 exhibited a Pearson’s correlation coefficient of 0.46 ± 0.14, whereas HA-Arf6/Q67L showed slightly higher colocalization (R = 0.59 ± 0.05), based on analysis of 30–35 cells three independent experiments. These data indicate moderate spatial proximity without evidence of direct co-trafficking or sequestration of BST-2.

To assess whether Arf6 expression influences overall BST-2 abundance, whole-cell lysates from cells transfected with vector control, HA-Arf6, or HA-Arf6/Q67L were analyzed by immunoblotting with anti-BST-2 and anti-actin antibodies. BST-2 levels were comparable across all conditions, and densitometric quantification normalized to actin confirmed the absence of significant changes (Figure 7B). Surface expression of BST-2 was further evaluated by flow cytometry. HeLa cells were co-transfected with GFP and either vector control, HA-Arf6, or HA-Arf6/Q67L, and surface BST-2 levels were quantified in GFP-positive cells using an antibody recognizing the extracellular domain of BST-2. Flow cytometric analysis revealed no significant difference in surface BST-2 expression among groups (Figure 7C), indicating that Arf6 activation does not affect BST-2 trafficking to the plasma membrane.

Collectively, these findings demonstrate that Arf6, similar to Arf1, does not alter the subcellular distribution, total abundance, or surface expression of BST-2. Thus, BST-2 is not regulated through Arf6-mediated membrane trafficking pathways.

## 4. Discussion

This study demonstrates a critical role for the small GTPase Arf1 and its regulatory machinery in controlling late stages of the HIV-1 replication cycle. We show that dynamic cycling between the GTP- and GDP-bound states of Arf1 is essential for efficient trafficking of the HIV-1 Gag polyprotein to the plasma membrane and for productive virion release. Disruption of this cycle—either through constitutive activation (Q71L) or inactivation (T31N)—impairs Gag membrane association and reduces particle production. These findings underscore the requirement for precisely coordinated Arf1 activity during viral particle assembly and reinforce the broader concept that small GTPases act as molecular switches governing vesicle-mediated trafficking.

Arf1 is a well-characterized regulator of intracellular membrane transport, particularly at the Golgi apparatus and early endosomes, where it recruits coat proteins such as COPI, modulates lipid composition, and contributes to membrane curvature [43,44,45,46,47]. Although its cellular functions are well defined, relatively few studies have addressed the role of Arf1 in retroviral replication [23,48]. Our data provide direct evidence that Arf1 activity influences the subcellular localization of HIV-1 Gag, a key determinant of viral assembly. Membrane flotation assays (Figure 2) and confocal imaging (Figure 1 and Figure S1) show that altered Arf1 cycling disrupts Gag membrane association and prevents its accumulation at the plasma membrane, thereby reducing particle release. Notably, Arf1 exhibits only modest colocalization with Gag (Figure 1), suggesting that its effects may be indirect. We propose that Arf1 modulates the trafficking environment by influencing membrane identity or lipid composition at the TGN or recycling endosomes, thereby promoting efficient Gag sorting and delivery. Alternatively, Arf1 may regulate the formation or distribution of phosphoinositide lipids such as PI(4)P or PI(4,5)P_2_—essential determinants of Gag membrane binding [49,50,51]. This model is consistent with the established role of Arf1 in recruiting lipid-modifying enzymes that shape organelle-specific lipid profiles [52,53], and with evidence that Gag preferentially interacts with PI(4,5)P_2_ at the plasma membrane [24].

A major insight from this work is the identification of AGAP1, an Arf1-specific GTPase-activating protein, as a negative regulator of HIV-1 assembly. AGAP1 promotes GTP hydrolysis on Arf1, converting it to the inactive GDP-bound state [39]. Overexpression of AGAP1 markedly reduced HIV-1 particle release and redistributed Gag to intracellular compartments, phenocopying the effects of the GDP-locked Arf1/T31N mutant. Conversely, a GAP-deficient AGAP1 mutant (R599K) did not affect Gag trafficking or particle production (Figure 3 and Figure S2), confirming that AGAP’s catalytic activity is required for its regulatory role. These findings support a model in which spatial and temporal control of Arf1 inactivation—likely mediated by AGAP1 at specific trafficking hubs—is critical for efficient Gag transport to the plasma membrane. Excessive or premature inactivation of Arf1 may divert Gag-containing vesicles into nonproductive pathways or impair vesicle formation.

Our results also suggest that GGAs and AGAP1 act sequentially within the Arf1-mediated trafficking pathway, particularly during clathrin-coated vesicle formation. GGAs serve as adaptor proteins that link activated Arf1 to clathrin, facilitating vesicle budding. Arf1 likely dissociates from GGAs before inactivation by AGAP1, consistent with reports indicating that GGAs and AGAP1 have overlapping Arf1-binding interfaces but act at distinct stages of the vesicle cycle [54,55]. In line with this model, previous studies have shown that GGA overexpression disrupts intracellular sorting and impairs retroviral particle release [23,48].

Notably, both persistent activation and excessive inactivation of Arf1 impair viral assembly, illustrating a “Goldilocks” requirement for balanced GTPase cycling. Similar paradigms have been described for Rab and Rho GTPases, whose regulated transitions between nucleotide-bound states ensures proper vesicle formation, transport, docking, and fusion [56,57,58,59,60,61]. Our findings extend this principle to Arf1 in the context of HIV-1 assembly and highlight the vulnerability of viral replication to perturbations in host membrane trafficking networks.

We further demonstrate that Arf6, a GTPase associated with endosomal membranes and the plasma membrane [62,63], disrupts HIV-1 Gag trafficking when constitutively activated. Expression of the GTP-locked Arf6/Q67L mutant redirected Gag from the plasma membrane to intracellular compartments and reduced particle production (Figure 4 and Figure S3). These results align with known roles of Arf6 in regulating actin remodeling, endosomal recycling, and lipid composition [64,65]—processes that directly intersect with late stages of viral budding. Arf6 has been implicated in regulating PI(4,5)P2 distribution at the plasma membrane; consistent with this, Arf6/Q67L induced Gag accumulation within PI(4,5)P2-enriched intracellular vesicles rather than at the cell surface [24]. Such sustained activation likely disrupts actin-dependent transport, alters lipid microdomains, or interferes with vesicle scission. WT Arf6 did not affect Gag localization (Figure 4), indicating that the observed defects reflect aberrant GTPase activity rather than overexpression.

Because membrane-trafficking pathways are highly interconnected, genetic perturbations of Arf-regulated processes may allow compensatory adaptation or secondary alterations over the course of analysis. Although siRNA-mediated knockdown of Arf1 or Arf6 inhibited HIV-1 NL4-3 release in HeLa cells [23], acute pharmacological inhibition provides an important complementary approach. In our prior work, however, short-term treatment (1 h) with the Arf-GEF inhibitor brefeldin A (BFA) did not inhibit HIV-1 Gag-Pol VLP release [66], suggesting that brief disruption of Arf1- or Arf6-dependent pathways is insufficient to affect late assembly, or that Gag trafficking kinetics lie outside the effective inhibition window. We also consider whether Arf perturbations broadly disrupt membrane trafficking, including clathrin-mediated endocytosis or the organization of essential plasma membrane lipids such as PI(4,5)P_2_. Our previous studies showed that dominant inhibition of clathrin-mediated endocytosis does not impair HIV-1 release, indicating that Gag delivery to the plasma membrane proceeds largely independently of clathrin-dependent pathways [40,66]. These results argue that the phenotypes observed upon Arf perturbation are unlikely to reflect a generalized collapse of clathrin-mediated trafficking. Although we did not directly examine PI(4,5)P_2_ distribution under Arf inhibition in this study, such analyses would provide an additional layer of mechanistic insight and will be incorporated into future work.

Importantly, our findings rule out the possibility that Arf1 or Arf6 regulate HIV-1 release through modulation of BST-2, a host restriction factor that tethers nascent virions at the cell surface [28,29,32]. Although both GTPases exhibited modest colocalization with BST-2-positive compartments, neither altered the total expression, surface abundance, or steady-state distribution of endogenous BST-2 (Figure 5, Figure 6 and Figure 7). Likewise, AGAP1 expression did not affect BST-2 localization (Figure 5). These results demonstrate that the release defects caused by Arf1 or Arf6 perturbation occur independently of BST-2 trafficking or function. Consistent with this interpretation, disruption of Arf activity in BST-2-negative HEK 293T cells impaired particle release, supporting a BST-2-independent mechanism. This conclusion aligns with the established role of HIV-1 Vpu as the primary antagonist of BST-2, promoting its downregulation and degradation [33,67,68,69,70,71]. Nevertheless, direct assessment of BST-2 antiviral activity under conditions of Arf1 or Arf6 dysfunction would further strengthen this conclusion and remains an important direction for future investigation.

Taken together, our findings demonstrate a requirement for Arf1 cycling during the late stages of the HIV-1 life cycle. Arf1 activity regulates Gag trafficking, membrane association, and particle release, and these processes are highly sensitive to perturbation in GTPase activity. AGAP1 functions as a key negative regulator by modulating Arf1 inactivation, while constitutive Arf6 signaling similarly disrupts Gag trafficking, underscoring the broader importance of Arf family GTPases in retroviral assembly. Future studies will be needed to identify the downstream effectors of Arf1 and Arf6, define the membrane compartments that support Gag trafficking, and explore whether pharmacological targeting of Arf-regulatory pathways may offer new strategies for inhibiting HIV-1 replication.

## Data Availability

All data supporting the findings of this study are included within the article and its Supplementary Materials. Additional raw data and reagents are available from the corresponding author upon reasonable request.

## Supplementary Materials

The following supporting information can be downloaded at: https://www.mdpi.com/article/10.3390/v18010011/s1, Figure S1: Arf1 regulates HIV-1 Gag subcellular localization. Figure S2: AGAP1 modulates HIV-1 Gag subcellular localization. Figure S3: Arf6 regulates the subcellular localization of HIV-1 Gag.
