# Supplementary material for: Arf GTPases Define BST-2-Independent Pathways for HIV-1 Assembly and Release
Source: Viruses. 2025 Dec 20;18(1):11. doi: 10.3390/v18010011 (PMC12846463; doi:10.3390/v18010011)
Supplement: Supplementary file 1 [file viruses-18-00011-s001.zip › viruses-3946166-supplementary.pdf]

Figure S1

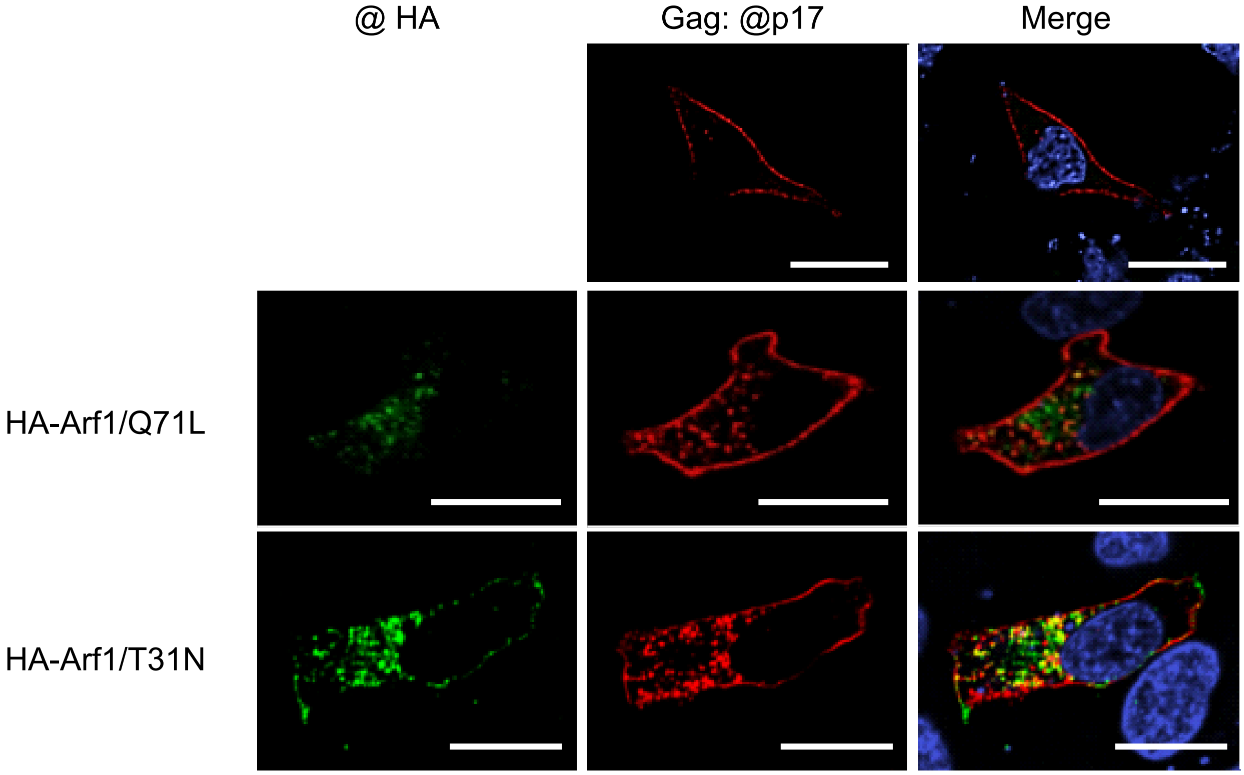

Figure S1. Arf1 regulates HIV-1 Gag subcellular localization. HeLa cells were co-transfected with HIV-1 Gag alone (top row), Gag with HA-Arf1/Q71L (middle row), or Gag with HA-Arf1/T31N (bottom row). At 24 h post-transfection, cells were fixed, permeabilized, and stained with anti-p17 (to detect Gag) and anti-HA antibodies. HA-tagged proteins are shown in green (left panels), Gag in red (middle panels), and merged images in yellow (right panels). Scale bars, 20 μm.

Figure S2

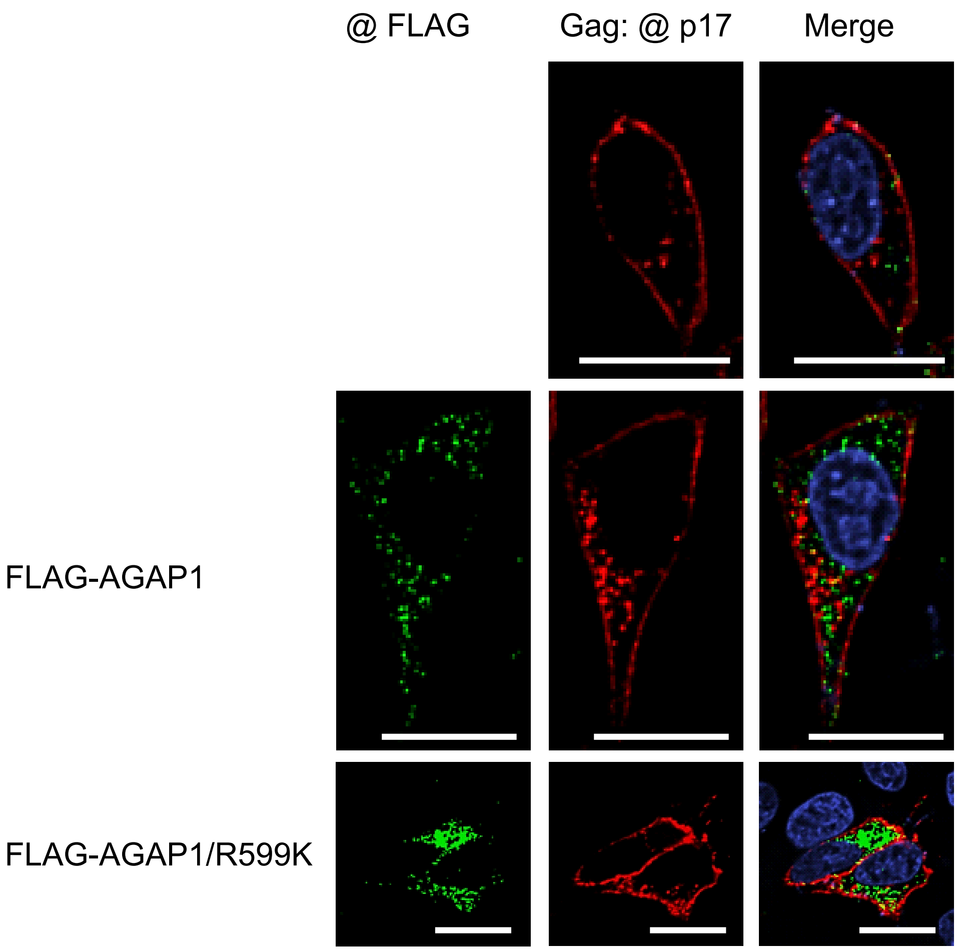

Figure S2. AGAP1 modulates HIV-1 Gag subcellular localization. HeLa cells were co-transfected with HIV-1 Gag alone (top row), Gag with FLAG-AGAP1 (middle row), or Gag with FLAG-AGAP1/R599K (bottom row). At 24 h post-transfection, cells were fixed, permeabilized, and stained with anti-p17 (to detect Gag) and anti-FLAG antibodies. FLAG-tagged proteins are shown in green (left panels), Gag in red (middle panels), and merged images in yellow (right panels). Scale bars, 20 μm.

Figure S3

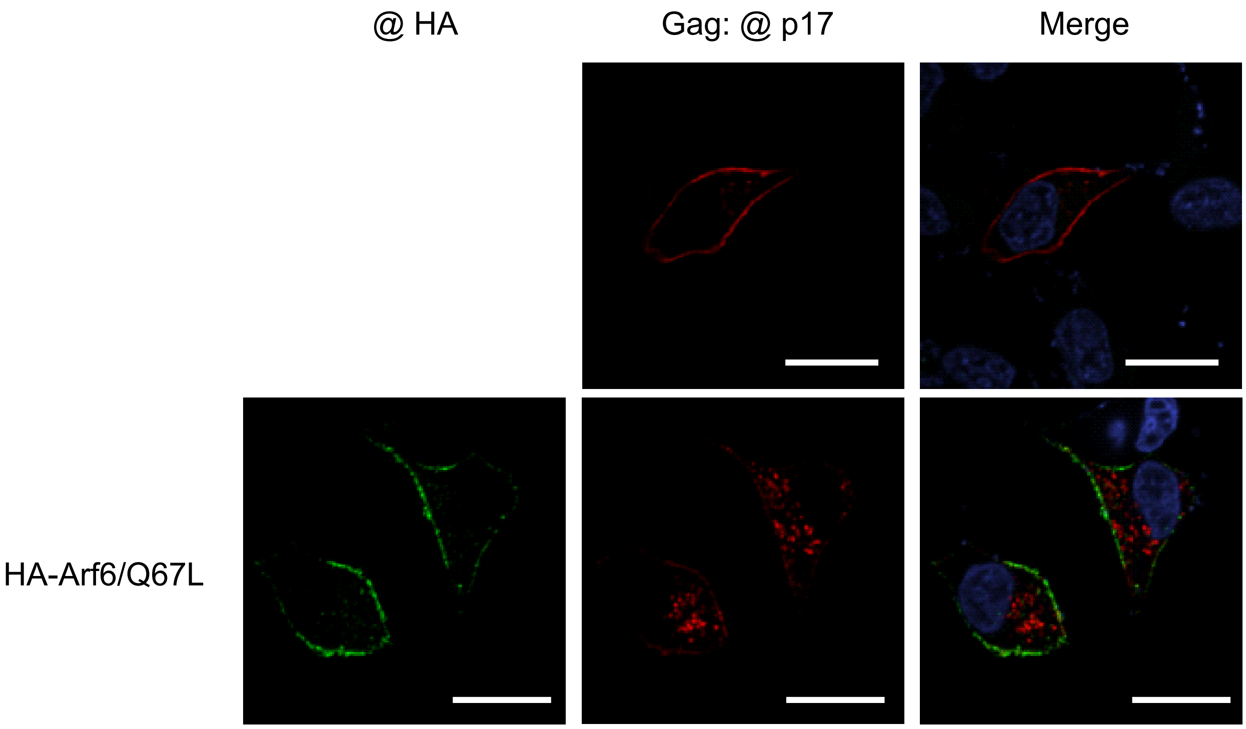

Figure S3. Arf6 regulates the subcellular localization of HIV-1 Gag. HeLa cells were co-transfected with HIV-1 Gag alone (top row), or Gag with HA-Arf6/Q67L(bottom row). At 24 h post-transfection, cells were fixed, permeabilized, and stained with anti-p17 (to detect Gag) and anti-HA antibodies. HA-Arf6/Q67L is shown in green (left), Gag in red (middle panels), and merged images in yellow
(right panels). Scale bars, 20 μm.
